# Paravertebral compartment syndrome after exercise: a case report

**DOI:** 10.1186/s13256-020-02535-1

**Published:** 2020-11-01

**Authors:** Tomofumi Ogoshi, Motoo Yoshimiya, Hiroshi Ichibakase, Takayoshi Kimura, Masafumi Kameoka, Hayato Yoshioka, Takahiro Ueda, Masato Homma, Shinpei Enokida

**Affiliations:** 1 Department of Emergency and Critical Medicine, Tottori University Hospital, 36-1, Nishichou, Yonago, Tottori, Japan; 2 Department of Orthopedics, Tottori University Hospital, 36-1, Nishichou, Yonago, Tottori, Japan

**Keywords:** Compartment pressure, Compartment syndrome, Creatine phosphokinase, Lumbar multifidus, Rower

## Abstract

**Background:**

Paravertebral compartment syndrome occurring without trauma is quite rare. We report a case of compartment syndrome that occurred after spinal exercises.

**Case presentation:**

A 23-year-old Japanese rower developed severe back pain and was unable to move 1 day after performing exercises for the spinal muscles. Initial evaluation at a nearby hospital revealed hematuria and elevated creatine phosphokinase levels. He was transferred to our hospital, where magnetic resonance imaging revealed no hematoma but confirmed edema in the paravertebral muscles. The compartment pressure measurements were elevated bilaterally. Despite his pain being severe, his creatine phosphokinase levels were expected to peak and decline; his urine output was normal; and surgery was undesirable. Therefore, we opted for conservative management. The next day, the patient’s compartment pressure diminished, and his pain levels decreased to 2/10. After 5 days, he was able to walk without medication.

**Conclusions:**

We present a rare case of compartment syndrome of the paravertebral muscles with good resolution following conservative management. We hope our case findings will help avoid unnecessary surgery in cases of paravertebral compartment syndrome.

## Background

Compartment syndrome (CS) is characterized by increased compartmental pressure after trauma or surgery. CS is almost always reported in a limb; occurrence in other parts of the body, such as the paravertebral muscles, is rare. Lumbar paravertebral CS was first described in a 1985 case report of a young man with postexertional back pain [1]. Paravertebral compartment syndrome has also been reported without local trauma in a few cases [2, 3]. The paravertebral compartment is enclosed by the fascia on the anterior, posterior, and lateral sides. Paravertebral compartment pressure has been shown to be affected by posture, the valsalva maneuver, and trunk extension effort over time [4]. We present a case of a patient with paravertebral CS without trauma or surgery.

## Case presentation

The patient was a 23-year-old Japanese man who was a medical student and belonged to a boating club. Two days before admission, he developed back pain after performing several repetitions of lifting 30-kg weight in a semicrouched position during a training session with the club. The pain was initially mild. On the day before admission, although he had mild back pain, he participated in both morning and evening training sessions. After the evening session, the pain gradually intensified. He waited to see whether the pain would resolve, but he was transported to a nearby hospital by ambulance at midnight because the back pain had worsened, and he found it difficult to move.

Because of the combined symptoms of back pain at rest, hematuria, and a calculus detected in the ureter by computed tomography (CT), he was diagnosed with a urinary calculus and treated with fluids and rest. However, his pain persisted without relief. The following morning, because blood tests revealed elevated creatine kinase (CK) (46,190 U/L) and lactate dehydrogenase (LDH) (1815 U/L) levels, CS was suspected. He was then transferred to our hospital.

Upon admission to our hospital, his condition was as follows: blood pressure, 131/81 mmHg; pulse, 88 beats/minute; oxygen saturation, 100% in room air; respiratory rate, 22 breaths/minute; and consciousness level on the Glasgow Coma Scale, E4V5M6. His abdomen was flat and soft, and no tenderness was observed. The erector spinae muscles were tense, with especially severe spontaneous pain and tenderness in the lumbar region. The skin in the lumbar region was free from blistering or other superficial lesions. No neurological abnormalities, such as muscle weakness or impaired sensation, were observed in the lower limbs. There were no other findings suggestive of CS in the limbs.

**Fig. 1:**
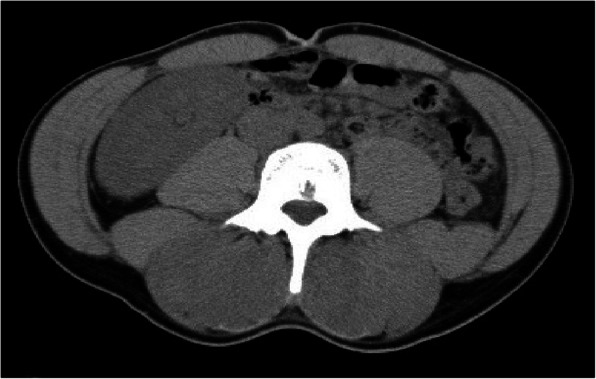
Abdominal computed tomography at admission. Swelling was noted in the paravertebral muscle

**Fig. 2:**
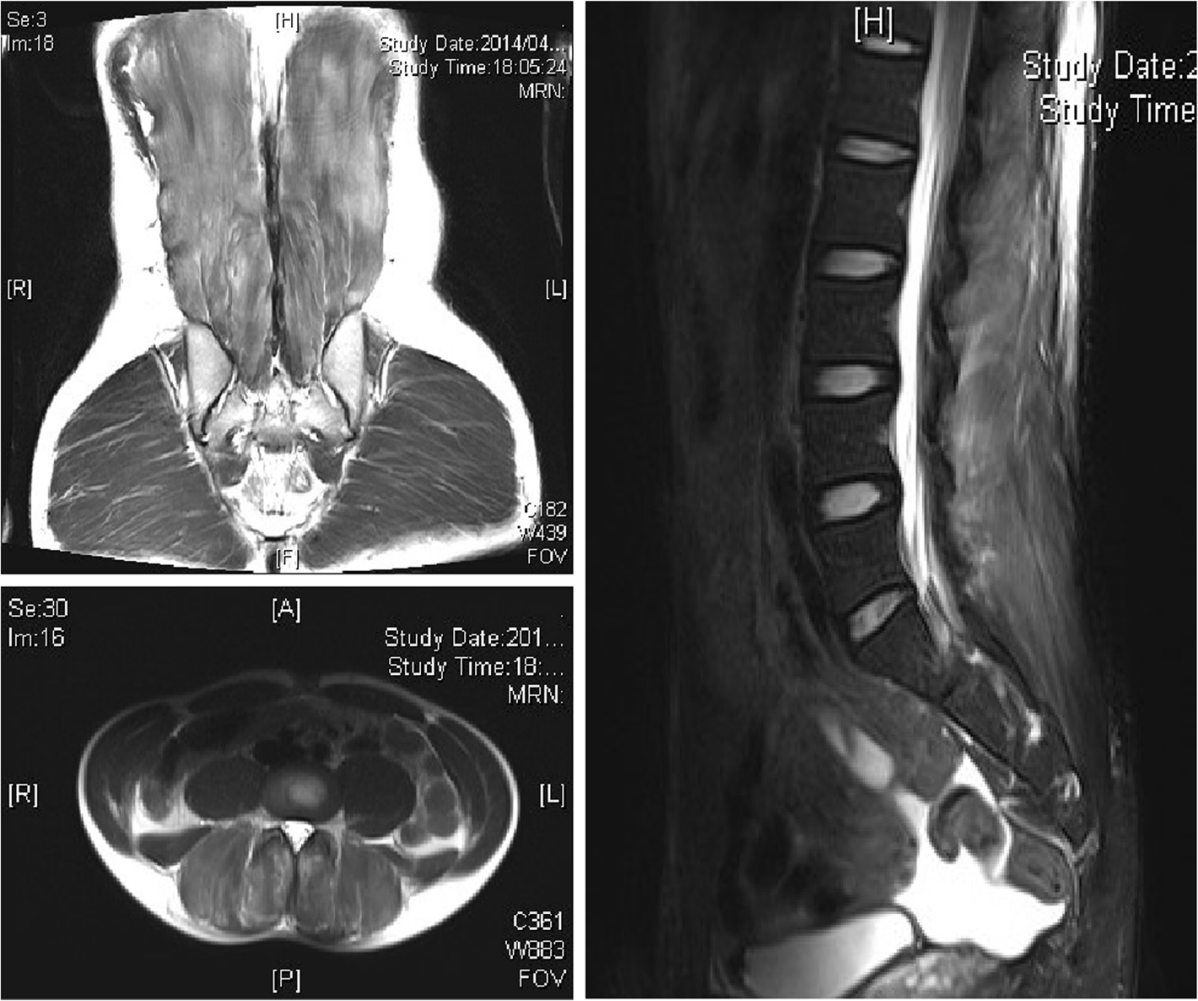
Magnetic resonance imaging at admission. The paravertebral muscles were edematous

**Fig. 3:**
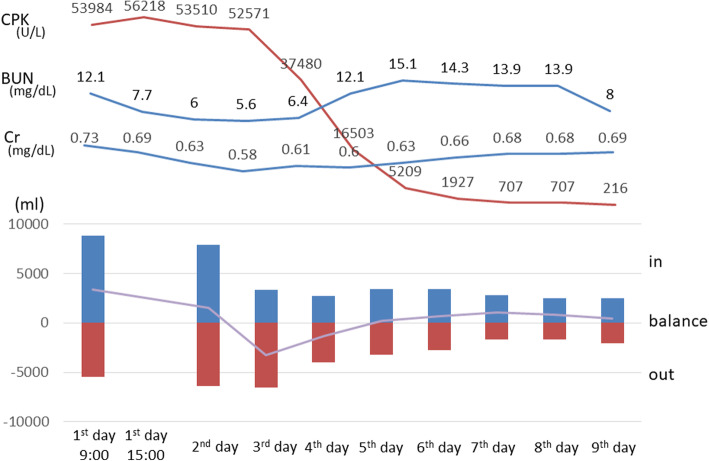
Creatine phosphokinase, blood urea nitrogen, and creatinine levels and water balance over time

Blood tests (Table 1) revealed increases in the serum levels of aspartate aminotransferase, 619 U/L; alanine aminotransferase, 157 U/L; LDH, 2050 U/L; creatine phosphokinase (CPK), 53,984 U/L; and myoglobin, 3756 mg/dl. Urinalysis revealed 3+ occult blood and an increased myoglobin level of 6500 mg/dl. Blood gas analysis (Table 2) revealed favorable oxygenation, and acidemia was not observed. Because the patient had a history of food allergy, plain CT was performed without contrast media. CT findings revealed swelling of the erector spinae muscles (Fig. 1). Although magnetic resonance imaging (MRI) revealed edematous lesions in the erector spinae muscles and low-intensity regions in the lumbar multifidus, there were no clear findings that indicated muscle necrosis (Fig. 2). Because paravertebral CS was suspected, compartment pressure was measured in the erector spinae muscles at the fourth and fifth lumbar levels, where the pain was severe. The pressure in the erector spinae muscles, measured at 3, 7, and 12 cm from the iliac crest, was elevated to 105 mmHg on the right side and 64 mmHg on the left side at 3 cm, and 95 mmHg and 48 mmHg, respectively, at 7 cm (Table 3). On the basis of these findings, paravertebral CS was diagnosed. A volume load of saline was administered, including 250 ml of 7% sodium bicarbonate, to alkalinize the urine and prevent progression to renal failure due to elevated urine myoglobin levels. After the infusion of sufficient external fluid, administration of D-mannitol was started. Although nonsteroidal anti-inflammatory drugs (NSAIDs) were initially used for pain control, the pain could not be controlled, and continuous infusion of a fentanyl citrate injectable solution was initiated. Urine output was maintained at 1 ml/kg/hour with sufficient infusion (Fig. 3); no deterioration of renal function was observed, and pain was controlled by continuous infusion of a fentanyl citrate injectable solution. Thus, conservative management was selected.

**Table 1:** Laboratory data on admission

Parameter	Value	Parameter	Value
Na	144 mEq/L	WBC	8.8 × 10^3^/μl
K	4.5 mEq/L	RBC	4.32 × 10^6^/μl
Cl	105 mEq/L	Hb	12.3 g/dl
Ca	9.8 mg/dl	Hct	38.50%
BUN	12.1 mg/dl	Plt	177 × 10^3^/μl
Cr	0.73 mg/dl	Neutro	90%
eGFR	111.3 ml/minute/1.73 m^2^	Lymph	6%
T.pro	6.9 g/dl	Mono	4%
Alb	5.2 g/dl	Eosino	0%
T. bil	2.2 mg/dl	Baso	0%
D. bil	0.5 mg/dl	PT (seconds)	
AST	619 U/L	PT INR	1.1
ALT	157 U/L	APTT (seconds)	26.4
ALP	156 U/L	D-dimer	0.4 μg/ml
LAP	54 U/L		
γ-GTP	12 U/L		
CHE	274 U/L		
LDH	2050 U/L		
Amy	81 U/L		
CPK	53,984 U/L		
CK-MB	682 U/L		
CRP	0.09 mg/dl		
Glu	99 mg/dl		

*Abbreviations: ALP* Alkaline phosphatase, *ALT* Alanine aminotransferase, *APTT* Activated partial thromboplastin time, *AST* Aspartate aminotransferase, *BUN* Blood urea nitrogen, *CK-MB* Creatine kinase myocardial band, *CPK* Creatine phosphokinase, *CRP* C-reactive protein, *eGFR* Estimated glomerular filtration rate, *γ-GTP* γ-Glutamyl transpeptidase, *Hb* Hemoglobin, *Hct* Hematocrit, *INR* International normalized ratio, *LAP* Leukocyte alkaline phosphatase, *LDH* Lactate dehydrogenase, *Plt* Platelet count, *PT* Prothrombin time, *RBC* Red blood cell count, *WBC* White blood cell count, *AMY* Amylase, *GLU* Glucose, *CHE* Cholinesterase, *T. bil* Total bilirubin, *D. bil* Direct bilirubin, *T.pro* Total protein, *Neutro* Rate of neutrophils, *Lympho* Rate of lymphocytes, *Mono* Rate of monocytes, *Eosino* Rate of Eosinophils, *Baso* Rate of Basophils

**Table 2:** Blood gas analysis data upon admission

Parameter	Value
FiO_2_	0.21
pH	7.398
pCO_2_	40.4 mmHg
pO_2_	77.7 mmHg
Hb	13.4 g/dl
HCO_3_	24.4 nmol/L
Na	139 nmol/L
K	4.2 nmol/L
Glu	105 mg/dl
Lac	1 nmol/L

*Abbreviations: FiO*_*2*_ Fraction of inspired oxygen, *pCO*_*2*_ Carbon dioxide pressure, *pO*_*2*_ Oxygen pressure

**Table 3:** Compartment pressure of the spinal muscles

Position from the iliac crest	First day		Second day	
	Right	Left	Right	Left
3 cm	105 mmHg	64 mmHg	99 mmHg	51 mmHg
7 cm	95 mmHg	48 mmHg	55 mmHg	47 mmHg
12 cm	37 mmHg	13 mmHg	19 mmHg	32 mmHg

**Fig. 4:**
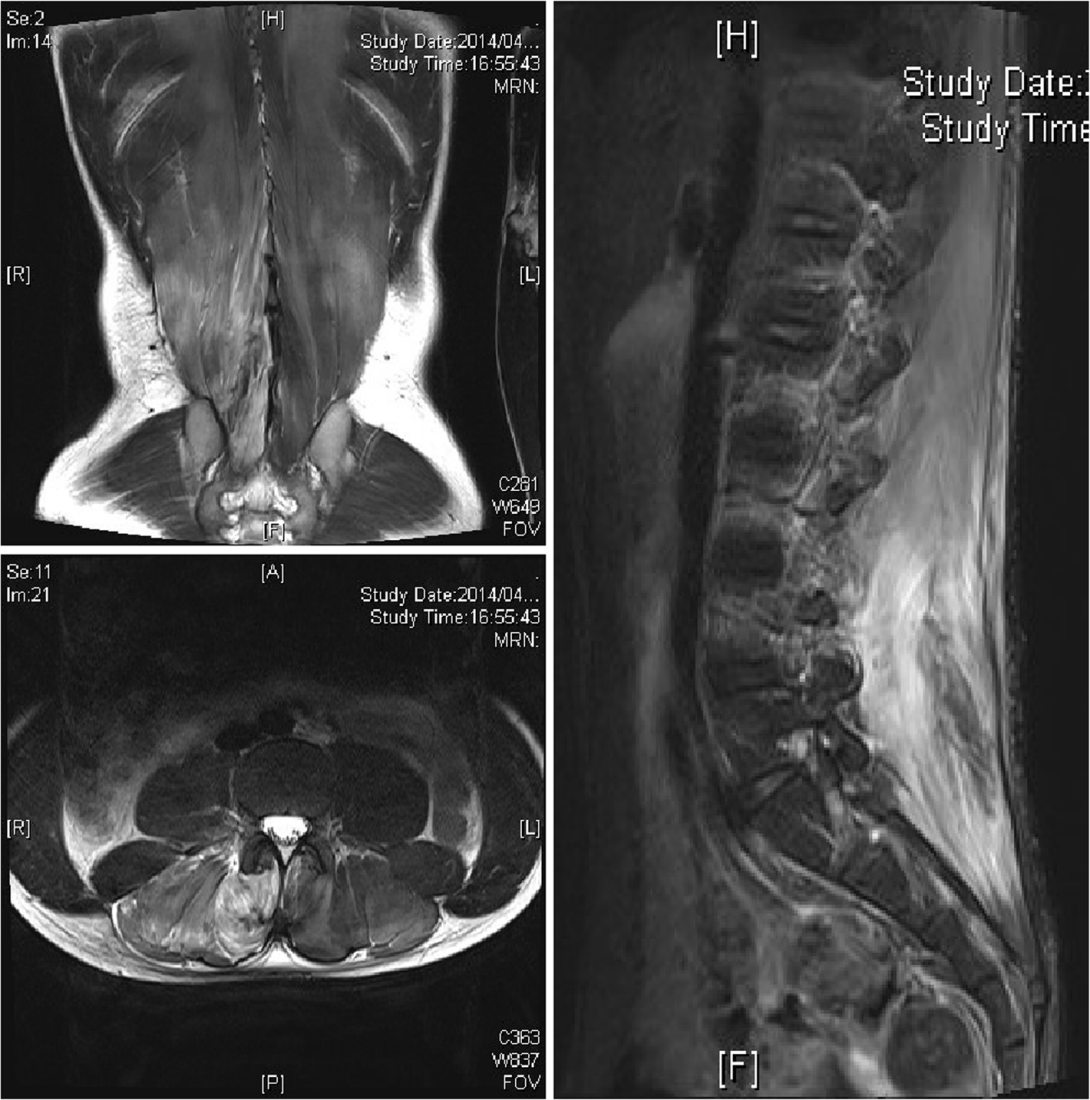
Magnetic resonance imaging after 7 days. Decrease in edema was seen on T2-weighted images

**Fig. 5:**
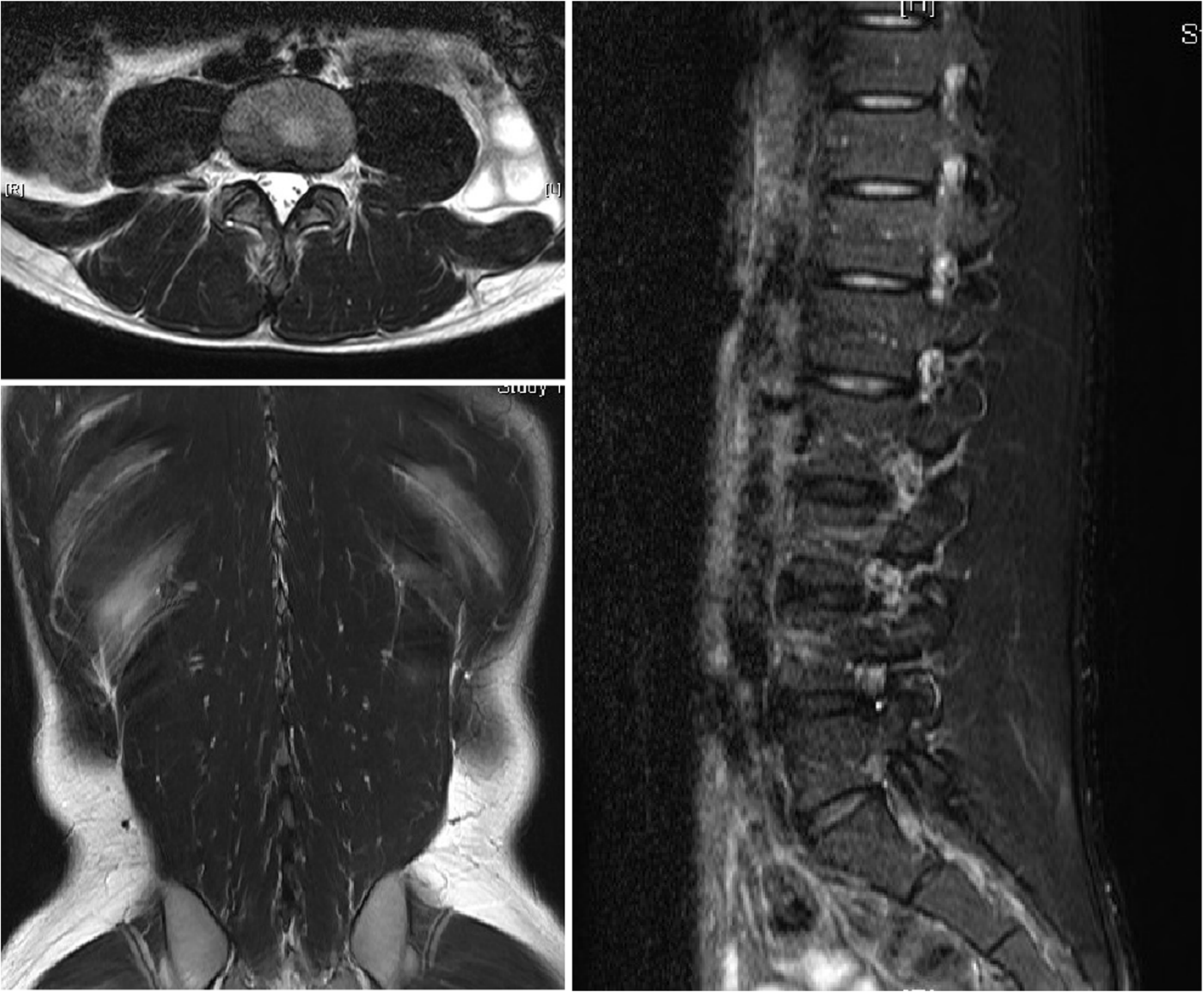
Magnetic resonance imaging after 3 months. No change was noted

**Fig. 6:**
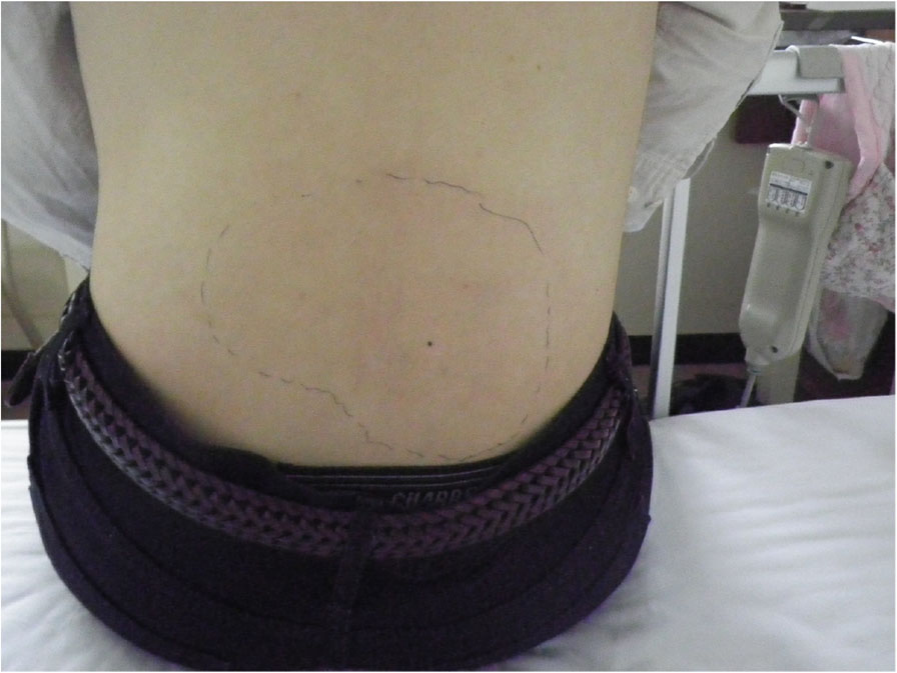
Area of discomfort persisted

The patient’s compartment pressure gradually decreased, and his CPK levels peaked on the first day of admission to the hospital (Fig. 3). The infusion of fentanyl citrate was subsequently discontinued and replaced with oral NSAIDs. Infusion was performed only with external fluid. The patient was able to get out of bed on hospital day 3 and returned to attending classes on hospital day 7. MRI performed on hospital day 7 revealed muscle necrosis in the right multifidus muscle and a reduction in edema in the left multifidus muscle. The area of high-intensity change in the right multifidus decreased on T2-weighted images (Fig. 4). On hospital day 10, the patient was able to perform activities of daily living without difficulty, despite discomfort in the right lumbar region, and was independently ambulatory. He was therefore discharged. MRI performed 12 weeks after onset revealed no signal intensity changes in the muscles (Fig. 5). Mild discomfort persisted in the marked lumbar region, as shown in Fig. 6. The patient was allowed to resume light sport activities at 24 weeks after onset because he did not feel any pain during his daily activities. Twelve months later, he could play tennis without pain. He reported mild discomfort in the lower back, the area of which decreased over time. The patient was scheduled to report for a follow-up at 24 months after the surgery. He consented to the publication of this case report.

## Discussion and conclusions

CS is frequently observed after trauma to the limbs. However, it rarely occurs in the erector spinae muscles, and the diagnosis is commonly delayed [5]. Our patient was initially diagnosed with a urinary calculus because of back pain, hematuria, and calculus detected using CT. Although measurement of CPK levels on the following day resulted in transfer of the patient to our hospital, the start of treatment was delayed by 1 day.

**Fig. 7:**
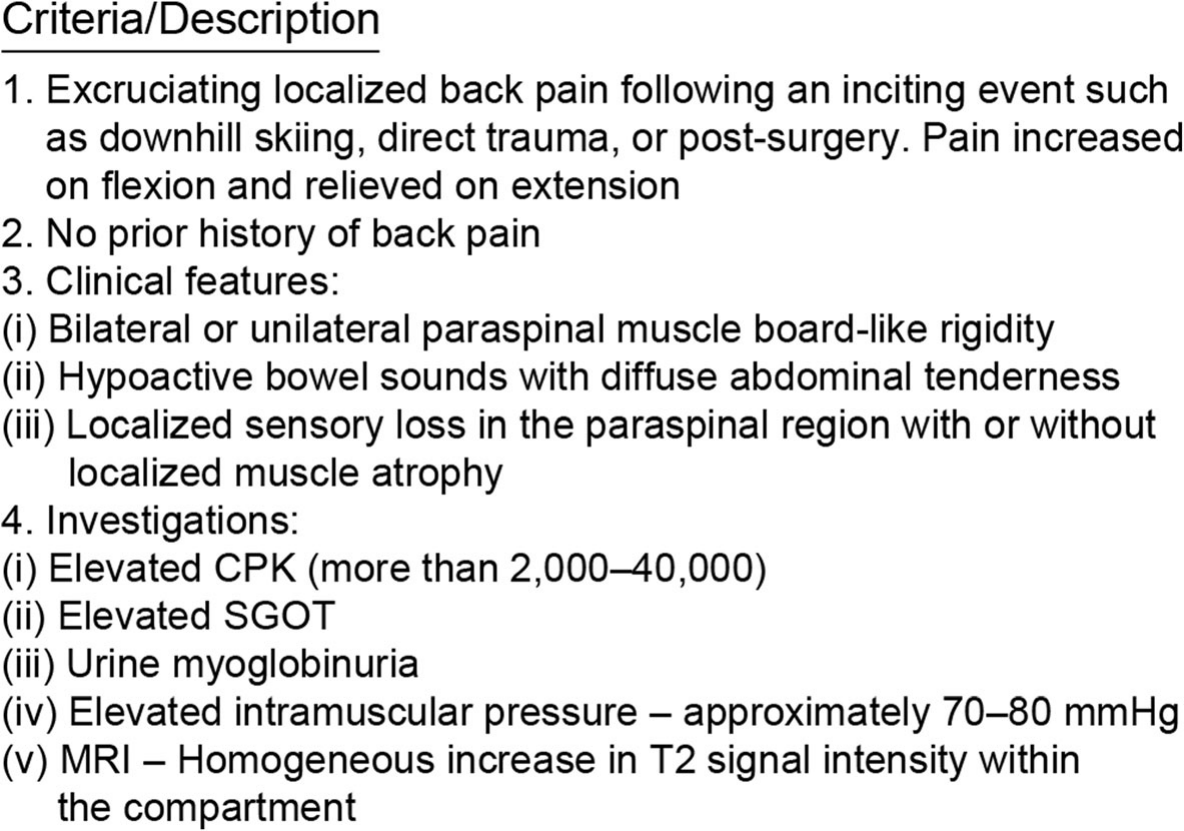
Diagnostic criteria for acute lumbar paraspinal compartment syndrome

Paravertebral CS is rare and difficult to diagnose. It has been characterized by the following symptoms in previous studies: severe tenderness, swelling, and rigidity in the lumbar region along with elevated CPK levels, according to Ferreira *et al.* [6]; abnormally high levels of CPK and blood myoglobin and sudden back pain associated with muscle edema depicted by imaging studies, according to King *et al*. [7]; dysesthesia in the lumbar region, according to Haig *et al*. [5] and Nathan *et al*. [8]; and increases in myoglobin, CPK, and compartment pressure with CT findings of muscle necrosis and ischemia, according to Mattiassich *et al*. [9]. Among the criteria for acute lumbar paravertebral CS (Fig. 7), our patient presented with increases in CPK, myoglobin, and compartment pressure, as well as tenderness and dysesthesia in the lumbar region, resulting in the eventual diagnosis of paravertebral CS. If characteristic findings such as severe pain, dysesthesia in the lumbar region, and elevated CPK levels are observed, CS should be suspected, and patients should undergo CT, compartment pressure measurement, and MRI. Moreover, in our patient’s case, we assume that muscle necrosis and hematoma could have been detected earlier using contrast-enhanced CT, although this was not performed due to the patient’s allergy to the contrast medium.

Regarding imaging findings, there are a few articles that mention CT. Plain CT is frequently performed for detailed examination of urinary calculi and the spinal canal in patients with sudden-onset back pain. In our patient, the diagnosis of paravertebral CS was delayed because a urinary calculus was detected at the previous hospital. Moreover, because there are few findings specifically suggestive of paravertebral CS, it appears to be difficult to diagnose the condition without performing contrast-enhanced CT. Regarding MRI findings, Ferreira *et al.* [6], Nathan *et al*. [8], and Mattiassich *et al.* [9] observed muscle edema on T2-weighted images. Mattiassich *et al*. also reported that muscle necrosis could be diagnosed on the basis of lack of gadolinium uptake [9]. In our patient, although MRI revealed extensive muscle edema, muscle necrosis was difficult to detect because contrast-enhanced imaging was not performed.

Although measurement of compartment pressure facilitates the diagnosis of paravertebral CS, normal pressure in the erector spinae muscles is not well established. Peck *et al.* reported that the normal pressure in weightlifting players is 3.11 mmHg in the supine position and 10.8 mmHg in the sitting position [2]. Moreover, Styf *et al.* reported that although muscle mass increases of 20% and compartment pressure increases from 8.5 to 14 mmHg are observed during exertional exercise, these values return to normal within 6 minutes after termination of exercise [10]. In the cases reported by Nathan *et al*., compartment pressure varied from 14 to 150 mmHg [8]. As shown in Fig. 7, one of the diagnostic criteria for CS is compartment pressure in excess of 70–80 mmHg. When this criterion is applied to the cases presented by Nathan *et al*., only one of their nine cases meets the criterion [8]. Thus, it seems difficult to diagnose CS on the basis of compartment pressure alone.

Of the therapeutic strategies employed for CS of the limbs, fasciotomy is indicated when the difference between diastolic blood pressure, which reflects the perfusion pressure in a muscle compartment, and intramuscular compartment pressure is within 20–30 mmHg. However, because this value is associated with a high false-positive rate and low specificity, continuous measurement of intramuscular compartment pressure for 30–60 minutes is reportedly preferable [11, 12]. The paravertebral compartment contains the sensory branch of the lumbar plexus but not the motor branch or the associated blood vessels. For paravertebral CS, the indications for surgical treatment are as follows: increased compartment pressure by 10–30 mmHg or more compared with diastolic blood pressure or by 50–70 mmHg compared with normal blood pressure, according to Whitesides *et al.* [4]; increases in CPK and compartment pressure, according to Haig *et al*. [5]; and uncontrollable pain and a sudden increase in CPK, according to Mattiassich *et al.* [9]. Our patient had severe back pain upon admission, which could not be controlled with NSAIDs but was controlled with fentanyl citrate. Moreover, his urine output was maintained with mannitol, sodium bicarbonate, and sufficient infusion. So, both CPK levels and compartment pressure peaked on the day of admission and subsequently decreased gradually. Due to these findings, conservative management was selected. Although the patient’s discomfort persisted in the lumbar region, his follow-up course appears to have been uneventful. Because Haig *et al*. indicated that follow-up MRI may reveal scarlike disuse changes in muscles affected by CS [5], we assume that further follow-up will also be needed for our patient.

In conclusion, we present a rare case of paravertebral CS. Despite high compartment pressures, conservative management resulted in an uneventful recovery with pain control and maintenance of urine output.

## Data Availability

All the data generated or analyzed during this study are included in this article and its supplementary information files.

## Abbreviations

ALP: Alkaline phosphatase
ALT: Alanine aminotransferase
APTT: Activated partial thromboplastin time
AST: Aspartate aminotransferase
BUN: Blood urea nitrogen
CK: Creatine kinase
CK-MB: Creatine kinase myocardial band
CPK: Creatine phosphokinase
CRP: C-reactive protein
CS: Compartment syndrome
CT: Computed tomography
eGFR: Estimated glomerular filtration rate
FiO_2_: Fraction of inspired oxygen
γ-GTP: γ-Glutamyl transpeptidase
Hb: Hemoglobin
Hct: Hematocrit
INR: International normalized ratio
LAP: Leukocyte alkaline phosphatase
LDH: Lactate dehydrogenase
MRI: Magnetic resonance imaging
NSAID: Nonsteroidal anti-inflammatory drug
pCO_2_: Carbon dioxide pressure
Plt: Platelet count
pO_2_: Oxygen pressure
PT: Prothrombin time
RBC: Red blood cell count
WBC: White blood cell count
